# Genetic relationships of European, Mediterranean, and SW Asian populations using a panel of 55 AISNPs

**DOI:** 10.1038/s41431-019-0466-6

**Published:** 2019-07-08

**Authors:** Andrew J. Pakstis, Cemal Gurkan, Mustafa Dogan, Hasan Emin Balkaya, Serkan Dogan, Pavlos I. Neophytou, Lotfi Cherni, Sami Boussetta, Houssein Khodjet-El-Khil, Amel Ben Ammar ElGaaied, Nina Mjølsnes Salvo, Kirstin Janssen, Gunn-Hege Olsen, Sibte Hadi, Eida Khalaf Almohammed, Vania Pereira, Ditte Mikkelsen Truelsen, Ozlem Bulbul, Usha Soundararajan, Haseena Rajeevan, Judith R. Kidd, Kenneth K. Kidd

**Affiliations:** 1 Department of Genetics, Yale University School of Medicine, New Haven, CT USA; 2 Turkish Cypriot DNA Laboratory, Committee on Missing Persons in Cyprus Turkish Cypriot Member Office, Nicosia, North Cyprus Turkey; 3 Dr. Fazıl Küçük Faculty of Medicine, Eastern Mediterranean University, Famagusta, North Cyprus Turkey; 4 Department of Genetics and Bioengineering, International Burch University, Sarajevo, Bosnia and Herzegovina; 5 Mendel Center for Biomedical Sciences, Egkomi, Nicosia Cyprus; 6 Laboratory of Genetics, Immunology and Human Pathologies, Faculty of Sciences of Tunis, University of Tunis El Manar, 2092 Tunis, Tunisia; 7 Higher Institute of Biotechnology of Monastir, Monastir University, 5000 Monastir, Tunisia; 8 Department of Biomedical Sciences, College of Health Sciences, Qatar University, Doha, Qatar; 9 Centre for Forensic Genetics, Institute of Medical Biology, UiT —The Arctic University of Norway, Tromsø, Norway; 10 School of Forensic & Applied Sciences, University of Central Lancashire, Preston, UK; 11 Ministry of Interior of Qatar, Doha, Qatar; 12 Section of Forensic Genetics, Department of Forensic Medicine, Faculty of Health and Medical Sciences, University of Copenhagen, 2100 Copenhagen, Denmark; 13 Institute of Forensic Science, Istanbul University, Istanbul, Turkey; 14 Center for Medical Informatics, Yale University School of Medicine, New Haven, CT 06520 USA

**Keywords:** Population genetics, Computational biology and bioinformatics

## Abstract

The set of 55 ancestry informative SNPs (AISNPs) originally developed by the Kidd Lab has been studied on a large number of populations and continues to be applied to new population samples. The existing reference database of population samples allows the relationships of new population samples to be inferred on a global level. Analyses show that these autosomal markers constitute one of the better panels of AISNPs. Continuing to build this reference database enhances its value. Because more than half of the 25 ethnic groups recently studied with these AISNPs are from Southwest Asia and the Mediterranean region, we present here various analyses focused on populations from these regions along with selected reference populations from nearby regions where genotype data are available. Many of these ethnic groups have not been previously studied for forensic markers. Data on populations from other world regions have also been added to the database but are not included in these focused analyses. The new population samples added to ALFRED and FROG-kb increase the total to 164 population samples that have been studied for all 55 AISNPs.

## Introduction

In previous publications [1, 2] we reported on the increasing number of population samples from major continental regions that had been studied for the panel of 55 ancestry informative single nucleotide polymorphisms (AISNPs) [3]. In 2017 there were 139 population samples (representing over 8000 individuals) with data on these AISNPs; analyses indicated that nine biogeographic regions could be distinguished. Allele frequencies and sample sizes have been incorporated into two freely accessible online databases—the ALlele FREquency Database (ALFRED: https//alfred.med.yale.edu) and the Forensic Reference-Resource on Genetics knowledge base (FROG-kb: https//frog.med.yale.edu). Given this global resource, studies of additional populations, especially for geographic regions poorly represented in the current dataset, are likely to be informative on the newly studied regions as well as the global pattern of variation. Here, we describe the newest reference populations that have become available for this set of AISNPs. As more than half of these populations represent ethnic groups from Southwest Asia and the Mediterranean, a number of which have either not been studied before or else not in an integrated manner, we take this opportunity to present analyses focused on groups from this region of the world and selected populations from immediately surrounding geographical region.

From the start of the Neolithic until now, Southwest Asia has been intimately involved in human history and the genetic consequences of the human migrations from and through the region are of considerable interest. Yet populations in this geographical region and nearby areas such as North Africa and Central Asia have not been well represented in the two largest of the human diversity studies, the CEPH-HGDP panel [4] and the 1000 Genomes panel (http://www.1000genomes.org) [5]. Including our current report’s emphasis on Southwest Asia and nearby regions, 25 new population samples (2278 individuals) have been added to these databases representing several major geographical regions of the world. The total collection now includes 164 population samples with data based on over 10,000 individuals.

## Materials and methods

### New population samples

The 25 new populations studied for the 55 AISNPs are listed in Table 1 [6–12]. Ten of these new reference populations and the data analyzed were obtained from recent publications; the other 15 population samples and data are collected or provided by co-authors of this study as indicated in Table 1. Informed consent was obtained for all newly collected population samples. The sample sizes (*N*), the laboratories generating the data, and the typing methods employed, along with the sample unique identifier (UID) in ALFRED are all included in Table 1 for the data being reported here. Supplementary Table S1 lists the 164 different population samples now available representing the diverse ethnic groups and biogeographic regions studied for these 55 AISNPs. The populations in Supplementary Table S1 are organized by geographic region; the table includes the sample size (2*N*), and the unique sample identifier in the ALFRED database for looking up the description of each sample. The three character population abbreviations employed in various figures in this report are also found in Table S1.

**Table 1:** The 25 new reference populations in FROG-kb for the 55 AISNP panel

Geographical region and population sample	Sample size (*N*)	Sample UID in ALFRED	Data sources and typing methods {footnote#}	Genotypes, frequencies available
Africa				
Southern Tunisians	96	SA004637U	{1}^a^	Both
Somalis	98	SA004636T	[6] {4}^b^	Both
Europe				
Norwegians	200	SA004650P	{2}^a^	Both
Danes	142	SA004635S	[6] {4}^b^	Both
Basques, Spain	108	SA004454R	[7]^c^	Both
Greek Cypriots	96	SA004645T	{1}^a^	Both
Southwest Asia				
Qatari	158	SA004651Q	{3}^a^	Both
Arabs, N. Iraq	130	SA004641P	{1}^d^	Both
Chaldeans, N. Iraq	22	SA004638V	{1}^a^	Both
Kurds, N. Iraq	148	SA004640O	{1}^d^	Both
Shabaks, N. Iraq	9	SA004643R	{1}^a^	Both
Syriacs, N. Iraq	125	SA004644S	{1}^d^	Both
Turkmen, N. Iraq	129	SA004642Q	{1}^d^	Both
Yazidis, N. Iraq	148	SA004639W	{1}^d^	Both
Turkish	88	SA004633Q	[8] {4}^b^	Both
Iranians	93	SA004634R	[8] {4}^b^	Both
East Asia				
Chengdu Tibetans	63	SA004624Q	[9]^c^	Both
Liangshan Tibetans	33	SA004616R	[9]^c^	Both
Qinghai Tibetans	25	SA004625R	[9]^c^	Both
Yi (Liangshan, Sichuan)	48	SA004626S	[9]^c^	Both
Japanese (Honshu)	49	SA004525Q	[10]^c^	Frequencies
Okinawa Japanese	47	SA004526R	[10]^c^	Frequencies
South America				
Afro-Ecuadorian	29	SA004509S	[11]^c^	Frequencies
Ecuadorian mestizo	67	SA004519T	[11]^c^	Frequencies
Kichwa (Ecuador)	66	SA004510K	[11]^c^	Frequencies

1. Genotypes generated at Kidd Lab employing standard TaqMan assays used previously for 55 AISNP panel [2, 3]. The seven population samples from Northern Iraq were collected by Mustafa Dogan, International Burch University; DNA extracted from buccal swab samples by Hasan Emin Balkaya at the Turkish Cypriot DNA laboratory. The Greek Cypriot samples were collected and DNA was extracted by Pavlos I. Neophytou, Mendel Center for Biomedical Sciences Nicosia. Southern Tunisians were collected via buccal swabs by Lotfi Cherni and colleagues at the University of Tunis el Manar

2. Genotypes provided by and the individual samples were collected by Nina Mjølsnes Salvo and colleagues, UiT—The Arctic University of Norway. Typing method: Illumina/Verogen ForenSeq DNA Signature Prep Kit on the MiSeq FGx Forensic Genomics System [12]

3. Genotypes provided by and the individual samples were collected by Sibte Hadi and colleagues, University of Central Lancashire. Typing method: Illumina ForenSeq DNA signature panel [12]

4. Genotypes for Danes, Somalis, Turkish, and Iranians provided by and the individual samples were collected in Denmark by Vania Pereira, Ditte M. Truelsen, Helle S. Mogensen, Maryam S. Farzad, Torben Tvedebrink, Claus Børsting, and Niels Morling, University of Copenhagen. All four samples consist of unrelated individuals collected in Denmark; the individuals from Somalia, Turkey, and Iran are immigrants to Denmark. Typing method: ThermoFisher Precision ID Ancestry panel

^a^Indicates population samples and SNP data reported for first time in this study

^b^Indicates population samples reported initially in a previous publication as cited here in the data source column of this table. SNP data in this study was supplied by co-authors; see footnote #4

^c^Indicates SNP data and population samples employed in this study that derive from publications as cited here in data source column of this table

^d^Indicates population samples initially reported in a previous publication [13] for Y-chromosome data only. The autosomal SNP data in this study has not been reported previously

Samples were collected primarily within the geographic bounds of an ethnic group’s home region but some were collected elsewhere (see Table 1 footnotes and citations). Individuals were self-identified as belonging to a specific ethnic group and reported the same ethnic group for their known ancestors. Supplementary Table S2 details the geographical distribution for the self-reported birthplaces of the individuals from Northern Iraq, belonging to seven different ethnic groups, all of which were residents in Northern Iraq at the time of sample collection. In a previous study [13] males from five of the seven N. Iraq groups (Arabs, Kurds, Syriacs, Turkmen, Yazidis) were typed for 17 STRP loci on the Y-chromosome and *in silico* haplogroup assignments were made. The patrilineal relationships of these groups are discussed there in light of these Y-haplogroup findings and comparisons are also made to what is known from the literature about other ethnic groups in the broader geographic region. The introductory section in Dogan et al. [13]. also provides additional historical and demographic information about the ethnic groups of ancient Mesopotamia and modern Iraq. Heated discussion occurs among some scholars about whether Chaldeans, Syriacs, and another ethnic group called Assyrians from N. Iraq are in fact all the same people, simply because they speak the same language—Syriac, a modern dialect of Aramaic [14]. Shabaks constitute a distinct ethnic community in N. Iraq; they speak a Kurdish dialect with many Arabic and Turkish loan words and they practice a strict form of Shi’a Islam based on a primary religious text, which is in the Turkmen language [15]. Supplementary Table S3 gives the geographic distribution for 96 Greek Cypriot samples (75 male, 21 female) for their self-reported residence at the time of sample collection.

### Statistical analyses

Every locus and population combination for which individual genotypes were available was tested for Hardy–Weinberg ratios on the assumption that each locus was a codominant di-allelic genetic system. Genotypes were examined to ensure that the alleles on the positive forward strand have been employed and the allele frequencies have been entered into the databases—ALFRED and FROG-kb.

Principal component analyses (PCA) of the population allele frequencies compared the similarities and differences among the populations. We used XLSTAT 2018 (http://www.xlstat.com/en/about-us/company.html) to calculate the PCs for the populations using their SNP frequencies. Table S4 summarizes the SNP allele frequencies for the 76 reference populations analyzed. These frequencies are also currently available in the static versions of the ALFRED and FROG-kb databases, but the future availability of these resources is uncertain because funding for them ended on 31 December of 2018.

The STRUCTURE software [16] provides a way of assessing how well a set of loci tested on multiple individuals can infer ancestry. We employed version 2.3.4 applying the standard admixture model assuming correlated allele frequencies. At each *K* value from 6 to 10, the program was run 20 times with 10,000 burn-ins and 10,000 Markov Chain Monte Carlo (MCMC) iterations. Table S5 contains the genotype profiles for 55 of 76 reference populations analyzed; this includes 3448 individuals. Genotypes for another 12 of the 76 populations are already in the public domain; these include a Basque group and 11 populations from the Thousand Genomes project. The remaining 9 of 76 populations are not in Table S5 due to various pre-existing agreements and/or a legal requirement of the country in which the DNA of participating individuals was collected. The five new populations in Table 1 for which genotypes are confidential include: Somalis (SMS), Norwegians (NOR), Danes (DNS), Turkish (TUR), and Iranians (IRD). Four other population samples that are confidential and are *not* among the new groups in Table 1 include: Tajiks (TJK), Kirghiz (KRG), and two Kazakh (KAZ, KZK) population samples.

Because we expect the Northern Iraqi populations to be closely related we also used the likelihood calculations in FROG-kb to examine how similar the likelihoods of populations would be for two Kurds as examples. As noted, FROG-kb now has all of the new population allele frequencies entered as reference population data. Two Kurds were selected to be examples for the 55 AISNP panel. We are aware that testing them against the entire sample from which they were chosen introduces a slight bias favoring finding them most similar to the Kurdish population. However, given the sample size involved (148 individuals) the bias is very small and can be ignored when using these as examples.

The calculation of likelihoods of ancestry for selected example individuals employed the function in FROG-kb for the Kidd lab 55 AISNP panel. The input is the genotype profile for each individual. For each population the calculation is the product of the frequencies of the genotypes of the input individual across all 55 loci. In the output the populations are ranked from highest to lowest likelihood.

The random match probability (RMP) for each population sample was computed assuming Hardy–Weinberg ratios. While RMP values will differ for each unique genotype, values have been calculated as the expected value based on the allele frequencies in each population. No correction was made for within-sample population structure since the focus is on distinct, well defined population samples representing global population structure.

Population tree diagrams are a common way to represent population relationships. The Neighbor Joining (NJ) method [17] is a commonly used algorithm to produce an approximately additive tree, i.e., a tree in which the pairwise genetic distances are additive across the segments (branches) of the tree connecting populations. Under this model the tree structure and the lengths of the segments can be represented as a series of linear equations that can be “solved” by least squares. Each tree structure gives a different set of linear equations. We use the tau genetic distance [18], which is theoretically additive in units of random drift. Unfortunately, the NJ approximation does not give an exact least squares estimate and when the NJ structure is evaluated by least squares some segments can have negative estimates of their length. Negative values of genetic drift should not exist and we prefer to find a tree that has all positive values as part of an exact least squares solution to the structure. We have used the LS search program [19] to search for a least squares estimate with all positive segments and minimum total length.

## Results

The SNP data collected for the 55 AISNPs by the various research groups employing several different methods are consistent in that the same alleles are being detected and the frequencies appear similar to other populations sampled in the same geographical region. For 20 of the 25 new populations (identified in Table 1) individual genotypes were available for this study either by direct contributions from research groups or by publication—usually in supplementary files at journal websites. Allele calls were standardized to the positive strand. No significant deviations from Hardy–Weinberg ratios were found beyond those expected by chance when multiple tests are carried out.

Allele frequencies in 164 population samples are now accessible in ALFRED and in FROG-kb for all 55 Kidd AISNPs. Some of the 55 SNPs have frequency data in ALFRED for >164 populations; those populations could not be included as reference populations in FROG-kb because they do not have frequencies for all 55 of the SNPs.

**Fig. 1:**
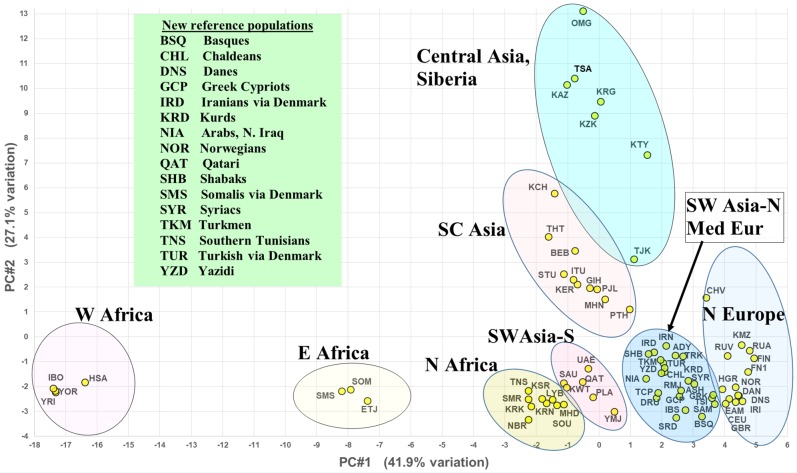
PCA results based on the 55 AISNP allele frequencies for 76 reference populations consisting of 43 groups from SW Asia–Europe and 33 selected populations in adjacent world regions. The 16 out of 25 new reference populations listed alphabetically in the inset box are from SW Asia, Europe, North Africa, East Africa

Figure 1 presents the PCA results on 76 reference populations for the first two principal components (PC), which account for 69% of the variation. The strong subgrouping of the populations in Fig. 1 clearly corresponds to the geographical proximity of the population samples. The first PC (41.9% of the variance) organizes the data from West Africa at one extreme to Northern Europe at the other extreme. The second PC (27.1% of the variance) separates South Central Asia and Central Asia from the rest, leaving a clear clinal organization of the North African to Southwest Asian to European populations. The third PC (only 7% of the variance) separates the Northern European populations from the Mediterranean and most Asian populations. The analyzed populations include all those with individual genotypes available from Southwest Asia, Europe, and North Africa. Selected outlier populations were added from adjacent geographical regions—East and West Africa, South Central Asia, and Central Asia. This dataset included 16 of the 20 new populations with genotypes. Four new populations from East Asia with available genotypes were not included in the analysis since the presentation was focused on the geographical regions where most of the newest reference populations were collected.

**Fig. 2:**
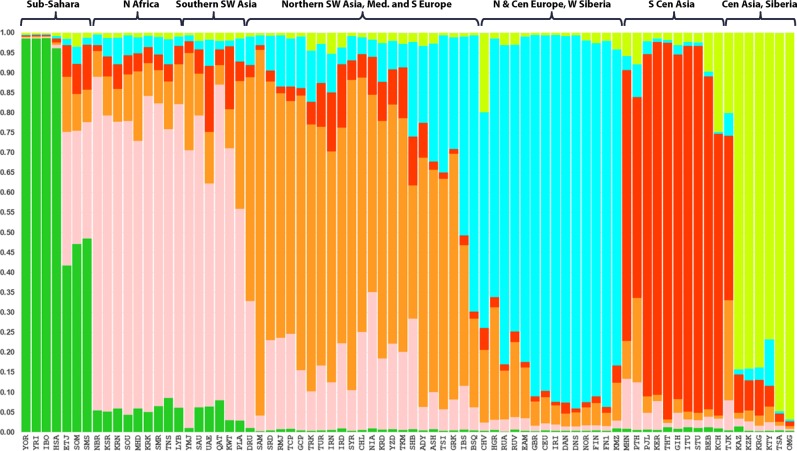
Estimated cluster membership bar plot via STRUCTURE for 43 reference populations in Southwest Asia—Europe and 33 selected populations in adjacent world regions—Africa, South Central Asia, Central Asia, Siberia. Displaying the highest likelihood run out of 20 runs at *K* = 6

The stacked bar plot in Fig. 2 shows the estimated cluster membership values as population averages for the highest likelihood result (out of 20 runs) for *K* = 6 of the STRUCTURE analysis of the same 76 populations as in the PCA analysis of Fig. 1. The dark green bar cluster on the left of the image includes the West African populations while the groups with the largest loadings for the pale tan cluster consists primarily of populations from North Africa and the southernmost part of SW Asia (corresponding mostly to the Arabian peninsula). The three East African populations (two Somali, Ethiopians) are more transitional showing moderate loadings on both the green and pale tan clusters. The populations with the largest orange bars are located primarily in Southern and Mediterranean Europe along with groups in the northern part of SW Asia (such as the new populations from N. Iraq, the Iranians, and the Turkish. The bright blue cluster encompasses populations from Northern Europe and a West Siberian group (the Komi Zyriane). The red bar cluster includes the South Central Asian populations from Pakistan and India. Finally, rightmost in Fig. 2 are the olive green population bars that include Central Asian/Siberian/Mongolian populations (i.e., Tajik, Khirgiz, Khazaks, Yakut, Khanty, Outer Mongolians, and Tsaatan).

### Population tree

The NJ tree structure, which contained 25 negative segments, was submitted as the initial input for the least squares algorithm and resulted in an exact least squares (LS) solution with both internal and terminal negative segments. The LS search program was used with several different input tree structures in addition to the NJ tree and a total of 387 different tree structures were evaluated by least squares. Eight slightly different tree structures among the highest ranked had no internal negative segments but all had 52 negative segments for the branches connecting populations to the backbone structure of the tree. A very small negative value for a segment connecting a population to the tree could be explained as sampling error. Unfortunately, not all the values were so small as to be explained away so simply.

## Discussion

### Population relationships

The graphical presentations based on analyses of the 55 AISNP panel show very strong geographical clustering of the 76 reference populations analyzed from Southwest Asia–Europe and the immediately adjacent areas. The PCA image (Fig. 1) of the first and second principal components shows eight distinct clusters emphasized by the color-circle overlays and text labeling. Four of these distinct clusters parse the core area very clearly—North Africa, southernmost SW Asia (mostly the Arabian peninsula), Northern SW Asia (Turkey, Iraq, Iran) and Mediterranean Europe, and Northern Europe. The STRUCTURE results (Fig. 2) show three main population clusters for the core region (Northern and Mediterranean Europe, Southwest Asia., North Africa); these correspond roughly to similar PCA groupings (Fig. 1). However, North African and the southern SW Asian area populations look somewhat more alike in the third STRUCTURE cluster in contrast to the PCA result where those areas appear more distinct. North Africa, of course, is only represented here by nine population samples from Tunisia and Libya; it would be interesting to see what relationships the 55 AISNPs would reveal if we had extensive population studies for them from Morocco, Algeria, and Egypt.

One way to think about the way that STRUCTURE defines clusters of individuals in an analysis is that it attempts to find Mendelian populations. From that perspective it becomes clear that the numbers of individuals in each input population is relevant as is the nature of the other populations. For example, a small population with a unique set of different allele frequencies can be absorbed into a large cluster if it is not too different relative to other possible clusterings. The deviation from Hardy–Weinberg ratios will not be that great. On the other hand, if the allele frequencies are different from all other groups, this small population can show membership in several clusters or cause the emergence of a distinct new cluster if the frequency differences are strong enough.

### Population tree

The large number of negative segments in all trees examined by both the inexact neighbor joining method and the exact least squares method argues strongly against the model of an additive genetic distance for these populations. Although one could find general groupings of populations with similarities to the clusters in the PCA analyses, we conclude that the underlying model of random genetic drift and a tree structure for relationships among the majority of these populations is invalid. The negative segments preclude a drawing. One possible explanation for the negative segments is that the populations are more similar than the model would predict and considerable gene flow would cause that to occur.

### Population variation

The random match probabilities (RMP) for the new populations in Fig. 4 are in the expected range given the values for the populations previously evaluated for these markers [20]. The values of RMP plotted are the expected or average values for the population based on the genotype frequencies. We note that RMP is a measure of the average heterozygosity of a panel and the variation among populations reflects their relative levels of within population variation for these particular SNPs. The values for the African populations are unusually low and this serves as a cautionary note to prevent over interpretation of the differences among populations. All we can confidently say is that differences exist for these markers. Indeed, we might have expected these SW Asian populations to have high levels of heterozygosity given the history of migrations known over the past few thousand years. We see that some populations that are more isolated have higher RMP levels as expected, e.g., Samaritans at 10^−9^, while others such as the Ethiopian Jews have among the lowest values of 10^−13^.

### Reference samples

A total of 164 population samples (10,356 individuals) now have allele frequencies on all of the 55 Kidd lab AISNPs. Sixteen of the newest reference populations have been studied via commercial kits from ThermoFisher Scientific or from Verogen that employ massively parallel sequencing. Researchers employing these resources will find the enhanced set of reference populations useful in a wide range of forensic, anthropological, and medical genetic studies. Both of the kits include the 55 Kidd AISNPs. The combined 55 Kidd and 128 Seldin [21] AISNP sets have 170 different AISNPs. The ThermoFisher Scientific kit includes 165 of these 170 AISNPs. Future analyses by the researchers who have conducted those studies may provide even more information on those populations’ relationships.

**Fig. 3:**
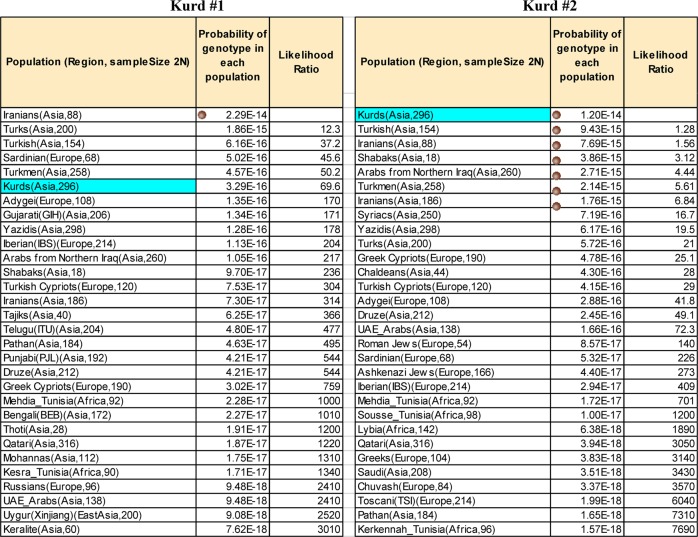
The 30 highest likelihoods calculated by FROG-kb for two Kurdish individuals based on the 55 AISNP panel and 164 current reference populations. The dot next to the value for the probability of genotype in each population identify results within one order of magnitude of the highest likelihood and therefore not significantly different

**Fig. 4:**
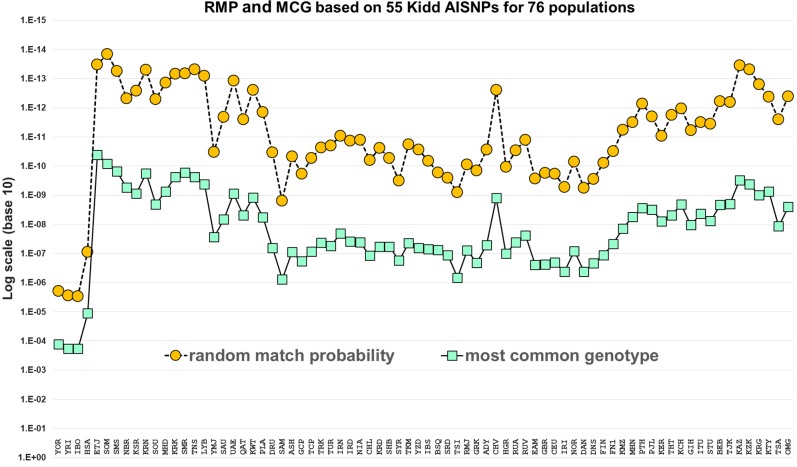
Random match probability and most common genotype frequency for each of 76 populations based on the 55 AISNP panel

The functionality in FROG-kb has been used to compute the relative likelihoods that two Kurdish individuals originate from each of the 164 reference populations now available in FROG-kb for the 55 AISNP panel (Fig. 3). These examples provide a clear demonstration that genotypes in this region of the world “overlap”. The genotype of Kurd #1 is most likely (among the reference populations) to occur in an Iranian population (Fig. 3). Based on the samples of the available reference populations, several other populations are more likely to be the origins of the genotype than the Kurdish population. However, although the Kurdish population is the sixth most likely to have generated this genotype, the likelihood ratio of Kurdish to Iranian is not highly significant, i.e., the ratios are less than two orders of magnitude apart. The genotype of Kurd #2 (Fig. 3) shows that the genotype is most likely to occur in the Kurdish population, based on the reference samples. Most interesting is that it is almost as likely to occur in several other reference populations that show no significant difference as potential origins of this genotype: six other populations have likelihood ratios separated by less than one order of magnitude. These include some of the same population samples that were seen among the not-highly significant reference populations for Kurd #1. These two examples illustrate the same need for caution in estimating ancestry using FROG-kb, or any statistically similar method, as discussed in previous publications [3, 22]. The highest likelihood is also the value for the random match probability for this individual that would be used in a forensic setting. In both cases the value is ~10^−14^, a meaningful value that would be especially valuable in combination with forensic STR markers. For these two examples the RMP values are smaller than the average for Kurds of between 10^−10^ and 10^−11^ (Fig. 4). The development of second-tier ancestry panels [23, 24] that can provide more refined differentiation of ancestry among closely related groups within geographical regions will be helpful in resolving at least some of the ambiguities of assignment that occur in ancestry inference panels designed for broader groupings of populations worldwide.

## Supplementary information

Suppl Tables S1,S2,S3

Suppl Table S4--dataset information

Suppl Table S5--dataset information
