# Supplementary material for: Genetic relationships of European, Mediterranean, and SW Asian populations using a panel of 55 AISNPs
Source: Eur J Hum Genet. 2019 Jul 8;27(12):1885–93. doi: 10.1038/s41431-019-0466-6 (PMC6871633; doi:10.1038/s41431-019-0466-6)
Supplement: Supplementary file 1 — Suppl Tables S1,S2,S3 [file 41431_2019_466_MOESM1_ESM.docx]

**Supplementary Material Tables S1, S2, S3**

**Genetic relationships of European, Mediterranean, and SW Asian populations using a panel of 55 AISNPs**

Andrew J. Pakstis^a^, Cemal Gurkan^b,c^, Mustafa Dogan^d^, Hasan Emin Balkaya^e^,

Serkan Dogan^d^, Pavlos I. Neophytou^f^, Lotfi Cherni^g,h^, Sami Boussetta^g^,

Houssein Khodjet-El-Khil^i^, Amel Ben Ammar ElGaaied^g^, Nina Mjølsnes Salvo^j^,

Kirstin Janssen^j^, Gunn-Hege Olsen^j^, Sibte Hadi^k^, Eida Khalaf Almohammed^k,l^,

Vania Pereira^m^, Ditte Mikkelsen Truelsen^m^, Ozlem Bulbul^n^, Usha Soundararajan^a^,

Haseena Rajeevan^o^, Judith R. Kidd^a^, Kenneth K. Kidd ^a^

**---------------------------------------------------------------------------------------------------------**

**Table S1.** The populations in ALFRED (https//alfred.med.yale.edu) with allele frequencies on all 55 Kidd ancestry informative SNPs. Populations are grouped by geographical regions of the world. The 25 newest reference population entries have yellow highlighting. More detailed population sample descriptions and citations can be found by searching in ALFRED with the UID.

| **World**  **Region** | **Population**  **Label ^a^** | **ALFRED**  **Sample**  **Unique Identifier**  **(UID)** | **ALFRED**  **Sample**  **Size**  **2N** | **Data source for**  **genotypes ^b,c^** | **Abbrev.**  **in**  **Figures** |
| --- | --- | --- | --- | --- | --- |
| Central Africa | Biaka | SA000005F | 140 | Kiddlab | BIA |
|  | Mbuti | SA000006G | 78 | Kiddlab | MBU |
|  | Lisongo | SA002770Q | 16 | Kiddlab | LIS |
| West Africa | Gambians | SA004243N | 226 | 1KG | GWD |
|  | Mende | SA004244O | 170 | 1KG | MSL |
|  | Esan | SA004248S | 198 | 1KG | ESN |
|  | Yoruba (Benin City) | SA000036J | 156 | Kiddlab | YOR |
|  | Yoruba (Ibadan) | SA004048Q | 216 | 1KG | YRI |
|  | Ibo | SA000099S | 96 | Kiddlab | IBO |
|  | Hausa | SA000100B | 78 | Kiddlab | HAS |
| East Africa | Luhya, Kenya | SA004046O | 198 | 1KG | LWK |
|  | Chagga | SA000487T | 90 | Kiddlab | CGA |
|  | Masai | SA000854R | 44 | Kiddlab | MAS |
|  | Sandawe | SA001773S | 80 | Kiddlab | SND |
|  | Zaramo | SA002586V | 80 | Kiddlab | ZRM |
|  | African Americans | SA004047P | 122 | 1KG | ASW |
|  | African Americans | SA000101C | 182 | Kiddlab | AAM |
|  | Ethiopian Jews | SA000015G | 64 | Kiddlab | ETJ |
|  | Somali | SA002138O | 40 | Kiddlab | SOM |
|  | Somalis via Denmark | SA004636T | 196 | V.Pereira | SMS |
| North Africa | Nebeur,Tunisia | SA004254P | 64 | Kiddlab | NBR |
|  | Kesra,Tunisia | SA004255Q | 90 | Kiddlab | KSR |
|  | Kairoun,Tunisia | SA004256R | 94 | Kiddlab | KRN |
|  | Sousse,Tunisia | SA004257S | 98 | Kiddlab | SOU |
|  | Mehdia,Tunisia | SA004258T | 92 | Kiddlab | MHD |
|  | Kerkennah,Tunisia | SA004259U | 96 | Kiddlab | KRK |
|  | Smar, South Tunisia | SA004260M | 130 | Kiddlab | SMR |
|  | Southern Tunisians | SA004637U | 190 | Kiddlab | TNS |
|  | Lybia | SA004261N | 142 | Kiddlab | LYB |
| SW Asia,  Med. Europe | Yemenite Jews | SA000016H | 146 | KiddLab | YMJ |
|  | Saudi | SA004393T | 208 | Kiddlab | SAU |
|  | U.A.E. Arabs | SA004394U | 138 | S.Hadi | UAE |
|  | Qatari | SA004651Q | 316 | S.Hadi | QAT |
|  | Kuwaiti | SA002765U | 32 | Kiddlab | KWT |
|  | Palestinian Arabs | SA002766V | 140 | Kiddlab | PLA |
|  | Druze | SA000047L | 212 | Kiddlab | DRU |
|  | Samaritans | SA000098R | 82 | Kiddlab | SAM |
|  | Turkish Cypriots | SA004333N | 120 | Kiddlab | TCP |
|  | Greek Cypriots | SA004645T | 190 | Kiddlab | GCP |
|  | Turkish, Istanbul | SA004310I | 154 | Kiddlab | TRK |
|  | Turkish via Denmark | SA004633Q | 186 | D.M.Truelsen | TUR |
|  | Iranians | SA004309Q | 88 | Kiddlab | IRN |
|  | Iranians via Denmark | SA004634R | 186 | D.M.Truelsen | IRD |
|  | Syriacs, N. Iraq | SA004644S | 250 | Kiddlab | SYR |
|  | Chaldeans, N. Iraq | SA004638V | 44 | Kiddlab | CHL |
|  | Arabs, N. Iraq | SA004641P | 260 | Kiddlab | NIA |
|  | Kurds, N. Iraq | SA004640O | 296 | Kiddlab | KRD |
|  | Yazidis, N. Iraq | SA004639W | 298 | Kiddlab | YZD |
|  | Turkmen, N. Iraq | SA004642Q | 258 | Kiddlab | TKM |
|  | Shabaks, N. Iraq | SA004643R | 18 | Kiddlab | SHB |
|  | Adygei | SA000017I | 108 | Kiddlab | ADY |
|  | Ashkenazi Jews | SA000490N | 166 | Kiddlab | ASH |
|  | Sardinians | SA002768X | 68 | Kiddlab | SRD |
|  | Roman Jews | SA000096P | 54 | Kiddlab | RMJ |
|  | Toscani | SA004057Q | 214 | 1KG | TSI |
|  | Greeks | SA002767W | 104 | Kiddlab | GRK |
|  | Iberians | SA004108N | 214 | 1KG | IBS |
|  | Basques | SA004454R | 216 | O.Garcia | BSQ |
| N. Europe | Chuvash | SA000491O | 84 | Kiddlab | CHV |
|  | Hungarians | SA002023H | 184 | Kiddlab | HGR |
|  | Russians, Archangel'sk | SA001530J | 68 | Kiddlab | RUA |
|  | Russians, Vologda | SA000019K | 96 | Kiddlab | RUV |
|  | Euro-Americans | SA000020C | 190 | Kiddlab | EAM |
|  | British | SA004050J | 182 | 1KG | GBR |
|  | Mixed Europeans | SA004250L | 198 | 1KG | CEU |
|  | Irish | SA000057M | 232 | Kiddlab | IRI |
|  | Danes (Copenhagen Blood Bank) | SA000007H | 102 | Kiddlab | DAN |
|  | Danes via Denmark | SA004635S | 284 | V.Pereira | DNS |
|  | Norwegians from Tromsø and Bodø | SA004650P | 400 | N.Mjølsnes Salvo | NOR |
|  | Finns | SA000018J | 72 | Kiddlab | FIN |
|  | Finns | SA004049R | 198 | 1KG | FN1 |
| Siberia, West | Komi-Zyrian | SA000489V | 94 | Kiddlab | KMZ |
|  | Khanty | SA000488U | 100 | Kiddlab | KTY |
| So.Cen. Asia | Mohannas | SA002139P | 112 | Kiddlab | MHN |
|  | Negroid Makrani | SA002137N | 56 | Kiddlab | MKR |
|  | Pathans | SA002873U | 184 | Kiddlab | PTH |
|  | Punjabi | SA004240K | 192 | 1KG | PJL |
|  | Keralites | SA001854S | 60 | Kiddlab | KER |
|  | Thoti | SA000077O | 28 | Kiddlab | THT |
|  | Gujarati | SA004246Q | 206 | 1KG | GIH |
|  | Telugu | SA004247R | 204 | 1KG | ITU |
|  | Sri Lankan Tamil | SA004241L | 204 | 1KG | STU |
|  | Bengali | SA004239S | 172 | 1KG | BEB |
|  | Kachari | SA000040E | 36 | Kiddlab | KCH |
|  | Hazaras | SA002140H | 60 | Kiddlab | HZR |
| Central Asia | Tajiks | SA004326P | 40 | C.-X.Li | TJK |
|  | Khazaks | SA002429R | 96 | H.Li | KAZ |
|  | Kazaks | SA004324N | 60 | C.-X.Li | KZK |
|  | Qinghai Tibetans | SA004625R | 50 | Z.Wang | QTB |
|  | Kirghiz | SA004325O | 60 | C.-X.Li | KRG |
| Siberia, East | Yakut | SA000011C | 102 | Kiddlab | YAK |
| East Asia | Tsaatan | SA004036N | 102 | Kiddlab | TSA |
|  | Outer Mongolians | SA004035M | 136 | Kiddlab | OMG |
|  | Inner Mongolians | SA002431K | 150 | H.Li | IMG |
|  | Inner Mongolians | SA004303K | 200 | L.Kang | MGL |
|  | Xibo | SA004323M | 58 | C.-X.Li | XBO |
|  | Tu | SA004319R | 52 | C.-X.Li | TUQ |
|  | Hui (Ningxia) | SA004304L | 198 | L.Kang | HUI |
|  | Koreans | SA000936S | 132 | Kiddlab | KOR |
|  | Koreans | SA004331L | 68 | C.-X.Li | KRS |
|  | Japanese | SA000010B | 112 | Kiddlab | JPN |
|  | Japanese, Tokyo | SA004060K | 208 | 1KG | JPT |
|  | Japanese (Honshu) | SA004525Q | 98 | H.Nakanishi | JHU |
|  | Okinawa Japanese | SA004526R | 94 | H.Nakanishi | OKN |
|  | Han (Shaanxi) | SA004305M | 200 | L.Kang | HNW |
|  | Han Chinese | SA004058R | 206 | 1KG | CHB |
|  | Han (Shandong) | SA004320J | 54 | C.-X.Li | HSH |
|  | Han (Yunnan) | SA004307O | 198 | L.Kang | HSW |
|  | San Francisco Chinese | SA000009J | 124 | Kiddlab | CHS |
|  | Taiwanese Han | SA000001B | 100 | Kiddlab | CHT |
|  | Han Chinese South | SA004059S | 210 | 1KG | HCS |
|  | Hakka | SA000003D | 86 | Kiddlab | HKA |
|  | Hakka (Meizhou) | SA004317P | 100 | C.-X.Li | HKK |
|  | Han (Guangzhou) | SA004314M | 80 | C.-X.Li | HGD |
|  | Han (Guangxi) | SA004318Q | 56 | C.-X.Li | HJX |
|  | Han (Guangdong) | SA004313L | 112 | C.-X.Li | TEO |
|  | Khamba Tibetans | SA002434N | 72 | H.Li | KHG |
|  | Tibetans | SA004322L | 100 | L.Kang | TBT |
|  | Chengdu Tibetans | SA004624Q | 126 | Z.Wang | CTB |
|  | Liangshan Tibetans | SA004616R | 66 | Z.Wang | LTB |
|  | Yi (Sichuan) | SA004626S | 96 | Z.Wang | LYI |
|  | Tibetans (Tibet) | SA004302J | 200 | C.-X.Li | TBS |
|  | Baima Dee | SA002432L | 84 | H.Li | BQY |
|  | Yi | SA004321K | 52 | C.-X.Li | YIS |
|  | Va (Yunnan) | SA004328R | 58 | C.-X.Li | VAY |
|  | Va (Myanmar) | SA004332M | 32 | C.-X.Li | VAB |
|  | Bai | SA004330K | 46 | C.-X.Li | BAI |
|  | Qiang | SA002433M | 80 | H.Li | QMR |
|  | Dai | SA004329S | 60 | C.-X.Li | DAI |
|  | Miao (Guangxi) | SA004316O | 50 | C.-X.Li | MAO |
|  | Miao (Guizhou) | SA004306N | 200 | L.Kang | HMO |
|  | Dong (Guangxi) | SA004312K | 56 | C.-X.Li | DNG |
|  | Jing (Guangxi) | SA004315N | 54 | C.-X.Li | JNG |
|  | Li | SA004308P | 200 | L.Kang | LIH |
|  | Chinese Dai | SA004238R | 186 | 1KG | CDX |
|  | Vietnamese | SA004249T | 202 | 1KG | KHV |
|  | Lao Long | SA001853R | 238 | Kiddlab | LAO |
|  | Cambodians | SA000022E | 52 | Kiddlab | CBD |
|  | Ami | SA000002C | 80 | Kiddlab | AMI |
|  | Atayal | SA000021D | 84 | Kiddlab | ATL |
|  | Malaysians | SA000097Q | 24 | Kiddlab | MLY |
| Pacific | Samoans | SA000072J | 22 | Kiddlab | SMO |
|  | Micronesians | SA000063J | 78 | Kiddlab | MCR |
|  | Papuans, New Guinea | SA000084M | 42 | Kiddlab | PNG |
|  | Nasioi speakers | SA000012D | 48 | Kiddlab | NAS |
|  | Aboriginals, Australia | SA004311J | 264 | Kiddlab | AUN |
| Greenland | Greenlanders | SA004396W | 178 | Morling | GRN |
| America, North | Plains Amerindians | SA000023F | 112 | Kiddlab | NPA |
|  | Southwest Amerindians | SA000025H | 104 | Kiddlab | SWA |
|  | Yavapai | SA004395V | 124 | Wendt, Budowle | YVP |
|  | Pima, Northern Mexico | SA000026I | 106 | Kiddlab | PMM |
|  | Maya,Yucatan, Mexico | SA000013E | 106 | Kiddlab | MAY |
| America, South | Guihiba speakers | SA000055K | 24 | Kiddlab | GHB |
|  | Kichwa | SA004510K | 132 | Santangelo | KWA |
|  | Afro-Ecuadorian | SA004509S | 58 | Santangelo | AEC |
|  | Ecuadorian Mestizo | SA004519T | 134 | Santangelo | ECM |
|  | Peruvians | SA004245P | 170 | 1KG | PEL |
|  | Quechua, Peru | SA000069P | 46 | Kiddlab | QUE |
|  | Ticuna | SA000027J | 134 | Kiddlab | TIC |
|  | Surui,Rondonia | SA000014F | 100 | Kiddlab | SUR |
|  | Karitiana | SA000028K | 114 | Kiddlab | KAR |
| **Table S1** footnotes  ^a^ Entries for the 25 newest population samples have yellow highlighting in this table. See also Table 1 for more details on sample collection, genotyping, and recent citations.  ^b^ See also population tables of [1,2] Pakstis et al. (2015, 2017) for information concerning sources for collection of previously contributed reference population samples.  ^c^ 1KG abbreviation stands for the 1000 Genomes Project--Phase 3. | | | | | |

**Table S2. Birth places of individuals sampled in seven populations of Northern Iraq.**

| Location | Kurds | Arabs | Syriacs | Turkmen | Yazidis | Chaldeans | Shabaks |
| --- | --- | --- | --- | --- | --- | --- | --- |
| Baghdad | 0 | 38 | 8 | 0 | 1 | 0 | 0 |
| Mosul | 3 | 22 | 64 | 0 | 74 | 0 | 0 |
| Erbil | 86 | 60 | 23 | 104 | 15 | 8 | 0 |
| Ramadi | 0 | 5 | 0 | 0 | 0 | 0 | 0 |
| Shaqlawa | 1 | 0 | 0 | 3 | 0 | 0 | 0 |
| Soran | 2 | 0 | 0 | 0 | 0 | 0 | 0 |
| Halabja | 1 | 0 | 0 | 0 | 0 | 0 | 0 |
| Sulaymaniyah | 39 | 0 | 0 | 1 | 0 | 0 | 0 |
| Said Sadiq | 2 | 0 | 0 | 0 | 0 | 0 | 0 |
| USA | 1 | 0 | 0 | 0 | 0 | 0 | 0 |
| Iran | 1 | 0 | 0 | 0 | 0 | 0 | 0 |
| Duhok | 0 | 0 | 0 | 2 | 25 | 0 | 1 |
| Kirkuk | 1 | 0 | 0 | 6 | 0 | 0 | 0 |
| Unknown | 18 | 11 | 36 | 21 | 40 | 14 | 8 |
| Total | 155 | 136 | 131 | 137 | 155 | 22 | 9 |

**Table S3. Residential locations of Greek Cypriots sampled in Cyprus.**

|  | **South Cyprus**  **5 administrative regions** | | | | |  |
| --- | --- | --- | --- | --- | --- | --- |
|  | Nicosia | Limassol | Larnaca | Paphos | South Famagusta | Totals |
| Individuals tested | 28 | 40 | 11 | 8 | 9 | 96 |
| Proportion tested | 0.292 | 0.417 | 0.115 | 0.083 | 0.094 | 1.000 |
| 2011 census  Greek Cypriots | 267266 | 188798 | 115299 | 58470 | 37565 | 667398 |
| 2011 proportion  Greek Cypriots | 0.400 | 0.283 | 0.173 | 0.088 | 0.056 | 1.000 |
